# How Reliable Are Sino-Nasal Cell Lines for Studying the Pathophysiology of Chronic Rhinosinusitis?

**DOI:** 10.1177/0003489414565003

**Published:** 2015-06

**Authors:** Stephen L. Ball, Monika I. Suwara, Lee A. Borthwick, Janet A. Wilson, Derek A. Mann, Andrew J. Fisher

**Affiliations:** 1 Institute of Cellular Medicine, Newcastle University, Newcastle upon Tyne, UK; 2 Freeman Hospital Department of ENT Surgery, Newcastle upon Tyne, UK

**Keywords:** rhinitis, sinusitis, cell-line, primary cells

## Abstract

**Background:**

Well-characterized cell lines represent useful scientific tools to study the pathophysiology of human disease. Chronic rhinosinusitis (CRS) is a very common condition, though the number of CRS cell lines is limited, as are data showing how closely they resemble primary cells.

**Methodology:**

Searches for available human cell lines were performed using the American Type Culture Collection (ATCC) and European Collection of Cell Cultures (ECACC). Identified cells were cultured and characterized with tinctorial and immunohistochemical staining and ELISA to assess their response to common, disease-relevant inflammatory stimuli. Carefully phenotyped CRS patients were recruited with informed consent. Primary nasal epithelial cell (PNEC) brushings were harvested, cultured, and compared to the available cell lines.

**Results:**

Searches identified 1 relevant CRS sino-nasal cell line, RPMI 2650. Cultured PNECs showed strong expression of epithelial markers while being negative for mesenchymal markers. However, RPMI 2650 cells show an atypical mixed epithelial/mesenchymal phenotype. When stimulated by pro-inflammatory ligands, PNECs responded in a dose-dependent manner, whereas RPMI 2650 cells showed limited response.

**Conclusions:**

The number and availability of cell lines to study the pathophysiology of CRS greatly underrepresent the disease burden. Additionally, the sole commercially available cell line appears to have a different phenotype and behavior to primary patient-derived cells. The development of further reproducible cell lines would be beneficial in our understanding of CRS.

## Introduction

Chronic rhinosinusitis (CRS) is one of the most common human conditions, with an estimated prevalence of 12.5%,^1^ greater than chronic back pain or diabetes.^2^ CRS generates annual direct health costs in excess $5.8 billion.^3^ The disease is often refractory to current pharmacological treatment with antibiotics and corticosteroids,^4^ leaving many patients facing the choice of surgery or persistent symptoms. Despite this lack of efficacy, medical treatment has demonstrated little change during an era that has seen significant innovation in surgical management. However effective surgery may now be in the short term, a relatively high disease recurrence rate persists.^5^ The lack of medical treatment options is however not surprising given our poor understanding of the pathogenesis of CRS. A concerted international effort utilizing a variety of methodological approaches, including clinical, in vivo, and in vitro cellular studies, is being pursued to improve our knowledge of chronic rhinosinusits. Within our CRS group we are currently focused on the association between the sinonasal epithelium and the sub-epithelial fibroblast layer and their roles in the persistent CRS inflammatory environment. To investigate this further, we have employed both patient-derived primary cultures of human sinonasal cells isolated from patients undergoing sinus operations and also sought to use immortalized cell lines for their inherent reproducibility. Cell lines are widely used to interrogate disease mechanisms throughout the body and act as rapid, effective laboratory models for hypothesis testing without the cellular heterogeneity or interindividual variability of patient-derived samples. To our surprise, the number of commercially available cell lines to study CRS is very limited and may not be representative of the parent tissue from within the sinonasal cavity.

## Materials and Methods

### Culture of RPMI 2650 Cells

Searches for commercially available cell lines were performed via the online catalogs of the American Type Culture Collection (ATCC) and European Collection of Cell Cultures (ECACC) using the search terms *nasal, sinus*, and *human*. Searches yielded 1 relevant cell line, RPMI 2650, which was purchased and grown in standard laboratory cell culture conditions. A vial of 2 × 10^6^ cells was cultured as per the suppliers instructions (ATCC) in Sigma EMEM (M2279, Sigma UK, Dorset, UK) with 1% non-essential amino acids (NEAA) (7145, Sigma UK), plus 100 iu/ml penicillin/streptomycin (P0781, Sigma UK), 50 ml fetal calf serum (FCS) (F9665, Sigma UK), and 2 mM L-Glutamine (G7513, Sigma UK). Cells were supplied at P26 and were amplified in T75 tissue culture flasks to generate sufficient cell numbers for our experiments. Cells were grown as submerged monolayer cultures in tissue culture flasks for stimulation experiments and on 13 mm coverslips for imaging.

### Culture of Primary Nasal Epithelial Cells

Participants undergoing elective operations for chronic rhinosinusitis according to the EPOS 2012 international consensus document^6^ were invited to participate in the study, with appropriate ethical and research governance approvals (UK National Research Ethics Service, REC reference 13/NE/0099). Primary nasal epithelial cells (PNECs) were harvested from the middle meatus by gentle passage with a cytology brush. Isolated PNECs were cultured in Lonza BEGM cell culture media (Lonza, CC-3171 & CC-4175) plus 100 iu/ml penicillin/streptomycin with media changed every 2 days until cells reached confluence.

#### Cellular Staining

Cells grown on 13 mm coverslips were fixed in 4% paraformaldehyde for 10 minutes and then washed with 1× phosphate buffered saline (PBS). Glycine (100 mM) was added to quench any remaining fixative. Cells were then permeabilized in 0.1% Triton-X100 (T8787, Sigma UK) in 1× PBS for 30 minutes and washed twice in PBS-0.2% Tween 20 (P1379, Sigma UK) and once further in 1× PBS. Cells were then either stained with H&E to assess cellular morphology or using immunocytochemical techniques for epithelial and mesenchymal markers. Primary antibodies were incubated at 4^o^C overnight (rabbit anti-human cytokeratin 17 Abcam Ab53707, Rabbit anti-human vimentin Abcam Ab92547), followed by fluorophore-conjugated secondary antibody (goat anti-rabbit TRITC conjugated Sigma T6778) at 1:100 dilution in 5% bovine serum albumin PBS-0.2% Tween 20 incubated for 90 minutes in the dark. Negative controls were performed with secondary only antibodies and matched IgG isotype negative controls to identify if there were nonspecific binding or background autofluorescence. Coverslips were mounted on slides with DAPI Vectashield (H1200, Vector Labs, Burlingame, California, USA), and images were captured with a Leica LSM 510 confocal microscope.

#### Cell Treatments

Monolayers of PNECs and RPMI 2650 cells approaching confluence were stimulated with CRS disease-relevant pro-inflammatory ligands: TNF-α 1 ng/ml, 5 ng/ml, 10 ng/ml (T0157, Sigma UK); LPS 0.1 µg/ml, 1 µg/ml, 10 µg/ml (L2630, Sigma UK); Poly I:C 50 µg/ml, a synthetic viral analogue (P9582, Sigma UK); and TGF-β 5ng/ml (T7924, Sigma UK) for 3 and 24 hours to determine if the cells were able to mount appropriate inflammatory responses. Untreated control cells without inflammatory ligands were cultured in parallel. Standard curves were performed as internal controls to ensure reproducibility between experiments. All cells were treated with identical conditions in triplicate repeats from the same batch of inflammatory ligands. Following stimulation, the conditioned media were harvested and the inflammatory response measured by the amount of IL-8 released into the culture media. Quantification was by ELISA for IL-8 protein as per manufacturer instructions (DY208, R&D Systems, Minneapolis, Minnesota, USA).

## Results

### Cellular Imaging

**Figure 1.**
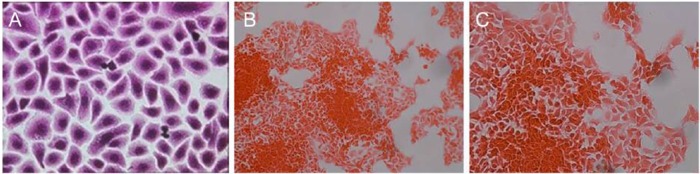
H&E stained cells grown in tissue culture conditions. (A) Primary nasal epithelial cells, magnification ×40. (B) RPMI 2650 cell line, note the growth in clusters and absence of a confluent monolayer, magnification ×20. (C) Magnification ×40.

**Figure 2.**
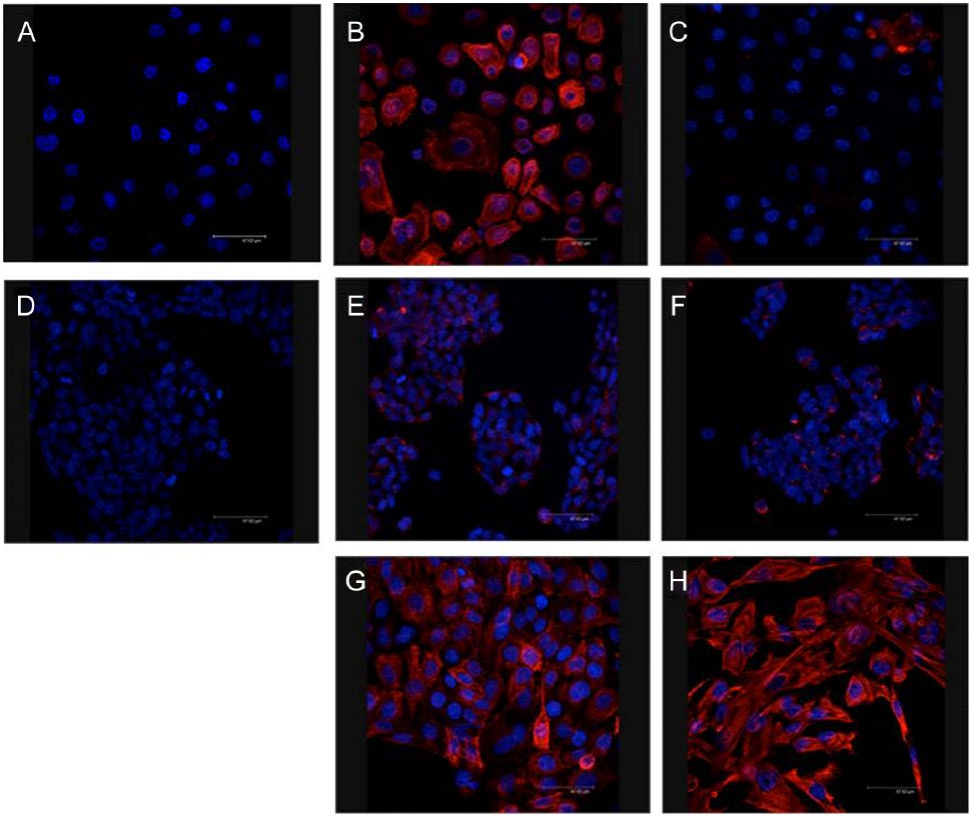
Fluorescent immunocytochemical images for epithelial and mesenchymal marker expression in primary nasal epithelial cells (PNECs) and RPMI 2650 cells. (A) Negative control showing only blue nuclear DAPI staining. (B) Positive PNEC staining with TRITC red cytokeratin 17 epithelial marker. (C) Negative mesenchymal PNEC staining with TRITC red vimentin marker. (D) Negative control showing only blue nuclear DAPI staining. (E) and (F) show RPMI 2650 cells weak TRITC red cytokeratin 17 epithelial and stronger TRITC red vimentin mesenchymal staining, respectively. (G) Positive control staining for cytokeratin 17 with HBE-14 human bronchial epithelial cell line. (H) Positive control staining for vimentin with MRC-5 lung fibroblast cell line. Magnification ×63.

Primary nasal epithelial cells were successfully grown as monolayers from participants undergoing surgery for CRS. H&E staining of these cells demonstrated a typical confluent cell culture monolayer (Figure 1A). Confocal microscopy showed that cells stained strongly positive for the epithelial marker cytokeratin 17 (CK-17, Figure 2B) and negatively for the mesenchymal or fibroblast marker vimentin (Figure 2C). In contrast, RPMI 2650 cells demonstrated different growth and cell-surface staining patterns compared to PNECs. RPMI 2650 cells grew in clusters and did not form typical epithelial confluent monolayers (Figures 1B and 1C). Immunocytochemical staining of RPMI 2650 cells showed an appearance that is atypical for epithelial cells, with stronger expression of mesenchymal fibroblastic markers (Figure 2 F) than epithelial markers (Figure 2E), which were only faintly positive. The immunostaining appearances, similar to the growth pattern, are not consistent for an epithelial cell line.

### Cell Stimulus Experiments

**Figure 3.**
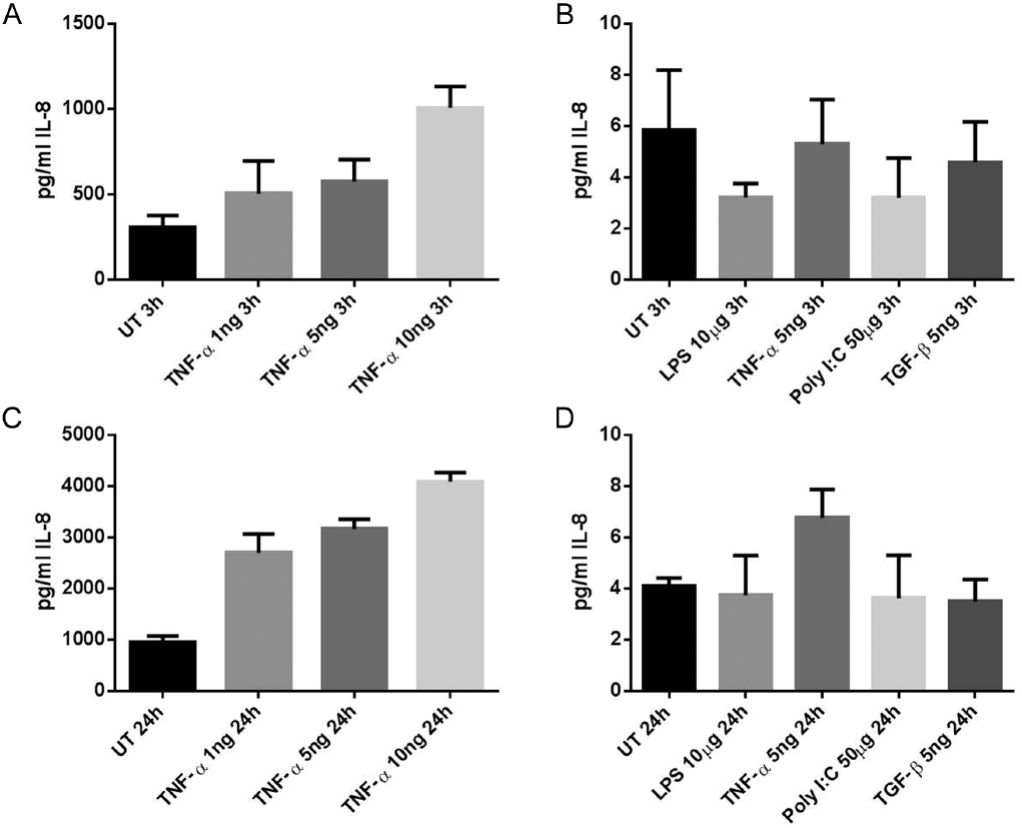
Amounts of IL-8 cytokine released into cell culture media following stimulation with disease-relevant chronic rhinosinusitis (CRS) inflammatory stimuli measured as protein content by ELISA. (A) Three-hour incubation of primary nasal epithelial cells (PNECs). (B) Three-hour incubation of RPMI 2650 cells. (C) Twenty-four–hour incubation of PNECs. (d) Twenty-four–hour incubation of RPMI 2650 cells. UT, untreated media only controls.

RPMI 2650 cells were additionally shown to produce very little inflammatory response as measured by IL-8 release to a range of ligands at 2 different time points. When compared to control untreated cells, tumor necrosis factor-α (TNF-α), lipopolysaccharide (LPS), poly I:C, and transforming growth factor-β (TGF-β) stimulated cells did not produce the expected inflammatory IL-8 response at either 3 or 24 hours (Figures 3B and 3D). The number of picograms IL-8 per milliliter detected was very low and never exceeded 10 pg/ml. In contrast, when PNECs were subjected to inflammatory stimuli with the CRS-relevant ligand TNF-α, they demonstrated a marked dose-dependent response, with IL-8 being produced up to 5000 picograms per milliliter (Figures 3A and 3C). Similar doses of TNF-α showed no response in RPMI 2650 cells.

## Discussion

Chronic rhinosinusitis is a very common condition that consumes significant health resources worldwide. There are vast direct health expenditures from the medical and surgical treatments for this condition. The mainstay of medical treatment has not changed from corticosteroids and antibiotics over many decades, at a time that has seen significant advances in the medical management of similar inflammatory conditions. Sinusitis remains the fifth most common indication for an antibiotic to be prescribed,^7^ yet antibiotics are not always effective. The position internationally of increasing antibiotic resistance and the slow progress in developing new antimicrobial agents^8^ illustrate further the unmet need for better CRS treatments. More modern, mechanistically focused anti-inflammatory and biological medications have been used in other chronic inflammatory conditions since the end of the twentieth century such as anti TNF-α therapy in rheumatoid arthritis^9^ and inflammatory bowel diseases.^10^ The prolonged use of corticosteroid medications is also not without risk. Although the majority of CRS steroid preparations are given topically rather than systemically, systemic absorption and suppression of the hypothalamo-pituitary axis does occur.^11^ The development of novel anti-inflammatory treatments for rheumatoid arthritis and inflammatory bowel diseases has followed from a greater understanding of the disease pathophysiology.^12^ Although such conditions are by no means completely characterized, advances in their detailed knowledge has translated into new therapies for patients. In many ways, CRS appears to show some similar pro-inflammatory and fibrotic disease characteristics,^13^ though these remain very early findings. Hopefully a more detailed understanding of the CRS disease mechanisms will lead to similar treatment advances for CRS patients.

Cell lines represent very useful laboratory tools to study the pathophysiology of human disease. Their use is widespread throughout all organ systems, which is reflected in the number and variety of available cell lines. The ECACC alone at present holds in excess of 40 000 cell lines available for study since its establishment in 1984.^14^ A search of the online catalog for ECACC, for example, offers researchers investigating pathology for similar inflammatory conditions in the lower airways with 97 possible cell lines available.^15^ Here we have identified only 1 commercially available cell line to study CRS, RPMI 2650. Unlike many other human conditions, there has been a paucity of well-validated reliable cellular models to study CRS. The only readily available commercial sinonasal cell line RPMI 2650 is often used in sinonasal studies,^16-19^ yet there is little published data about its relationship to sinonasal cells and its validity as a cellular model.

There are of course some shortcomings from the experimental approach that we have utilized to compare patient-derived PNECs with the sinonasal cell line RPMI 2650. First, we have grown both cell types in submerged culture rather than at an air-liquid interface to force differentiation. We chose this approach as in earlier air-liquid culture experiments we were not able to create a differentiated ciliated epithelium with RPMI 2650 cells (S Ball & co-authors, unpublished data) in agreement with previous work investigating the use of the cell line in nasal drug delivery studies.^20,21^ A major function of cells of the sinonasal cavity is in mucociliary clearance, which due to the nature of submerged culture could not be assessed here.

From our investigations we have shown that the sinonasal cell line RPMI 2650 is significantly different from patient-derived PNECs in terms of its cellular morphology, surface marker expression, and biological response to CRS disease-relevant inflammatory ligands such as TNF-α.^22-25^ While this is initially disappointing, it is perhaps not surprising when we investigate the origin of the cell line. It was derived from an anaplastic squamous cell carcinoma of the nasal septum in a 52-year-old male.^26^ Tumor cells were isolated from his metastatic pleural effusion and shown to grow as adherent nasal epithelial cells. The cell line has been proven to have a similar diploid karyotype to normal nasal epithelial cells.^27^ It has also been shown to have some similarity in terms of the expressed surface cytokeratins,^28^ and it produces a mucoid material that is visible on the cells apical surface.^26^ To date, the cell line has only been validated as a model to study the regulation of TGF-β biology in house dust mite–related allergic rhinitis.^29^ The neoplastic source of these cells perhaps explains the mixed epithelial and mesenchymal phenotype and growth pattern in cell culture. As a result, presently we would not recommend the use of RPMI 2650 as an in vitro cellular model for the sinonasal epithelium in CRS studies. In preference, we would advocate the use of primary, patient-derived nasal epithelial cells until such time that better validated sinonasal cell lines are established. The unmet disease burden from CRS would no doubt benefit from better CRS cell lines to aid our progress in understanding and treating this condition.
